# LDblockR: a cross-platform R toolkit for multi-format linkage disequilibrium analysis and integrated regional genomics visualization

**DOI:** 10.3389/fpls.2026.1991155

**Published:** 2026-10-26

**Authors:** Xiaofeng Song, Pengyu Chen, Fei Liang

**Affiliations:** 1 North Henan Medical University, Xinxiang, China; 2 Key Laboratory of Tropical Fruit Biology of Ministry of Agriculture, South Subtropical Crops Research Institute, Chinese Academy of Tropical Agricultural Sciences, Zhanjiang, China

**Keywords:** D′, GWAS, haplotype block, linkage disequilibrium, plant genomics, R package, R2, tag SNP

## Abstract

Linkage disequilibrium (LD) analysis is central to candidate-gene refinement, haplotype characterization, marker selection, and the interpretation of genome-wide association studies. However, commonly used LD programs often require format conversion, platform-specific binaries, or separate tools for LD calculation, block detection, regional annotation, subgroup comparison, and publication-quality visualization. We developed LDblockR, a pure-R, cross-platform toolkit that unifies regional LD analysis from VCF/VCF.GZ, HapMap, PLINK BED/BIM/FAM or PED/MAP files, and numeric 0/1/2 genotype matrices. LDblockR calculates dosage- or phased-haplotype-based r² and Lewontin’s D′, estimates haplotype frequencies for unphased diploid genotypes using an expectation-maximization procedure, and supports multiple block definitions, tag-SNP selection, LD-neighbor searches, LD decay, haplotype frequencies, population-group comparisons, and batch regional analysis. Its principal contribution is workflow integration and interoperability rather than a new LD estimator: a single R-native object model links heterogeneous genotype inputs, established LD statistics, regional annotation, and publication-ready visualization. In the bundled phased benchmark of 60 diploid samples and 42 variants (861 unique pairs), LDblockR agreed with LDBlockShow 1.41 to the three-decimal precision reported by LDBlockShow, with all r² and D′ values within 0.00051. We additionally analyze the official TASSEL/rTASSEL maize HapMap tutorial dataset, comprising 281 maize inbred lines and 3,093 genome-wide markers. After allele-consistency, missingness, and MAF filtering, 2,526 markers remain, including 428 SNPs on chromosome 1. Chromosome-wide dosage r² analysis covers 91,378 pairs; a highlighted 1-Mb interval contains 16 SNPs and 120 pairs. Independent pairwise calculations match the archived regional reference table to a maximum absolute difference of 5.01 × 10^−13^. This real-data example demonstrates regional LD analysis on crop genotypes with observed missing calls and allele-metadata conflicts. LDblockR therefore provides a transparent bridge between genotype input, regional LD analysis, and post-GWAS interpretation for plant association genetics and breeding.

## 1 Introduction

Linkage disequilibrium (LD), the non-random association of alleles at different loci, is a fundamental property of population genomic data and a key determinant of mapping resolution in association studies. Pairwise LD statistics such as r² and Lewontin’s D′ are routinely used to summarize local allelic correlation, define haplotype blocks, identify variants represented by tag markers, and interpret the genomic interval surrounding association peaks (Hill and Robertson, 1968; Lewontin, 1964; Gabriel et al., 2002). As genotyping density has increased, regional LD visualization has become a standard component of candidate-locus analysis in both human and plant genetics.

Several widely used tools address complementary aspects of this problem. Haploview established an influential framework for LD and haplotype visualization (Barrett et al., 2005), PLINK provides efficient genome-wide genotype processing and LD calculations (Purcell et al., 2007), and the R package LDheatmap enables flexible heatmap visualization within the R environment (Shin et al., 2006). More recently, LDBlockShow provided a fast command-line solution for producing LD heatmaps directly from VCF files while combining association statistics, genomic annotations, and subgroup analyses (Dong et al., 2021). These programs remain valuable, but practical plant-genomics workflows frequently involve heterogeneous input formats, particularly HapMap tables, PLINK files, VCF data, and custom genotype matrices. In addition, users often combine multiple programs or *ad hoc* scripts to move from genotype input to filtering, LD estimation, block definition, tag-SNP selection, LD decay, subgroup comparison, and publication-ready regional figures. Related ecosystems include TASSEL for association mapping and TASSEL-GBS for genotyping-by-sequencing pipelines (Bradbury et al., 2007; Glaubitz et al., 2014), PLINK 2 for scalable genotype processing (Chang et al., 2015), and Bioconductor tools for variant annotation (Obenchain et al., 2014).

Plant association studies provide a strong use case for integrated regional LD analysis. The maize association population was assembled to represent diversity in public-sector breeding programs, including historically important temperate and tropical materials as well as sweetcorn and popcorn lines; the original resource was described as 302 inbred lines (Flint-Garcia et al., 2005). Its population structure and breeding history matter because LD patterns are population- and locus-dependent, and association tests require appropriate control of relatedness and structure. The TASSEL tutorial genotype file used here is a reduced historical subset rather than a dense genome-wide panel. It demonstrates real crop-genotype ingestion, quality control, and regional LD summaries; interpretation of locus-level LD resolution is limited by its marker coverage. More generally, a reproducible tool should make it straightforward to inspect the local LD context around candidate associations while leaving association testing and population-structure correction to appropriate GWAS methods (Yu et al., 2006; Zhang et al., 2010; Zhou and Stephens, 2012).

Here, we present LDblockR, a pure-R package designed for regional LD analysis and integrated genomic visualization. LDblockR is not intended to replace optimized genome-scale engines or to introduce a new estimator of LD. Its methodological contribution is instead an integrated and reproducible workflow: heterogeneous genotype formats are normalized into a common R object and connected to established r²/D′ statistics, multiple haplotype-block definitions, tag-SNP and LD-decay analyses, subgroup comparison, genomic annotation, and publication-ready regional plots. We describe the software architecture, statistical implementation, validation against LDBlockShow, a real maize case study using the official TASSEL/rTASSEL tutorial dataset, practical scalability considerations, and the intended role and limitations of the package in plant genomic workflows. These design goals complement established association-mapping frameworks rather than replacing them (Yu et al., 2006; Zhang et al., 2010; Zhou and Stephens, 2012).

## 2 Method

### 2.1 Software design and requirements

LDblockR version 0.0.1 is implemented entirely in R and requires R ≥ 4.1.0. The package imports only the base/recommended graphics, grDevices, stats, and utils components and does not require compilation, Java, Bioconductor, or ggplot2. Consequently, the source package is intended to install on Windows, macOS, and Linux wherever a compatible R installation is available. The software is released under the MIT license. For indexed VCF files, an optional bcftools backend can be detected automatically and used for accelerated regional extraction; if bcftools is unavailable, LDblockR falls back to an internal streaming reader. BCFtools is a widely used toolkit for variant processing and regional queries (Danecek et al., 2021). The underlying file-processing conventions are compatible with established SAM/BAM and variant-processing ecosystems (Li et al., 2009; Danecek et al., 2021).

### 2.2 Input normalization and variant filtering

LDblockR accepts four major genotype representations: (i) plain or gzip-compressed VCF; (ii) standard HapMap text, including IUPAC heterozygous calls; (iii) PLINK binary BED/BIM/FAM and text PED/MAP files; and (iv) numeric genotype matrices coded as 0, 1, 2, or missing values, accompanied by a variant map. VCF is a standard representation for sequence variation (Danecek et al., 2011), whereas PLINK remains a common representation for genome-wide association and population-genetic analyses (Purcell et al., 2007). All readers normalize the input to a common ld_data object containing a samples-by-variants genotype matrix, variant metadata, sample identifiers, and—when recoverable from phased VCF—haplotype alleles. Input-format and filtering metadata are retained for downstream inspection. Variant filtering can be applied during input using minor allele frequency (MAF), missing-call fraction, heterozygous-call fraction, and Hardy–Weinberg equilibrium P-value thresholds. VCF input can additionally be restricted to PASS/unfiltered biallelic SNP records. Regional extraction accepts chromosome:start-end coordinates. When the number of retained variants exceeds a user-defined maximum, the workflow can either stop with an informative error or evenly thin variants for exploratory regional analysis; statistical export otherwise retains the complete filtered set. These input conventions also reflect the historical HapMap resource and current R/Bioconductor and PLINK ecosystems (International HapMap Consortium, 2003; Obenchain et al., 2014; Chang et al., 2015).

### 2.3 Pairwise r² and D′ estimation

For two biallelic loci A and B with derived-allele frequencies pA and pB and haplotype frequency pAB, LD is defined as D = pAB − pA pB. LDblockR reports r² = D²/[pA(1−pA)pB(1−pB)] and absolute standardized D′ = |D|/Dmax, where Dmax is determined by the allele frequencies and the sign of D. Both statistics are constrained to [0,1]. The r² implementation has three modes. In dosage mode, r² is calculated as the squared Pearson correlation between 0/1/2 genotype dosages using pairwise-complete observations. In haplotype mode, the squared correlation is calculated from the 0/1 haplotype matrix. In auto mode, complete phased haplotypes are used when all retained variants have sufficient phase information; otherwise dosage r² is selected. This design avoids treating unphased heterozygotes as if phase were known while retaining a direct haplotype statistic for phased data. For D′, directly observed haplotypes are used when a variant pair is completely phased. For unphased diploid genotypes, the four two-locus haplotype frequencies are estimated by expectation–maximization. Ambiguity from double heterozygotes is iteratively divided between coupling and repulsion configurations until the maximum frequency change falls below 10^−10^ or 1,000 iterations are reached. Optional 90% likelihood-weighted descriptive intervals for D′ are obtained by evaluating the two-locus genotype likelihood over a 0–1 D′ grid, normalizing the likelihoods as grid weights, and reporting the 5th and 95th percentiles. These summaries are not standard profile-likelihood intervals or coverage-calibrated frequentist confidence intervals. Pairwise calculations can be restricted by physical distance and minimum non-missing sample count. The allele-frequency dependence and finite-sample behavior of r² should be considered when interpreting values near rare-allele limits (VanLiere and Rosenberg, 2008).

### 2.4 Haplotype-block detection and tag-SNP selection

LDblockR implements five block strategies plus a no-block mode. The Gabriel-inspired method adapts the D′ interval-based classification concept introduced by Gabriel et al. (2002), using the package’s likelihood-weighted descriptive bounds rather than standard confidence intervals. Thresholds are configurable; the default classifies a pair as strong LD when the lower interval bound is ≥0.70 and the upper bound is ≥0.98, treats an upper bound <0.90 as evidence of recombination, and requires >95% of informative pairs in a candidate interval to be strong. The solid-spine method requires strong LD from each endpoint to all intervening variants. The strong-pair method defines a candidate block by the fraction of pairwise values exceeding a configurable r² or D′ threshold. The four-gamete method terminates blocks where all four haplotypes are observed above a minimum frequency. Finally, user-specified genomic intervals can be supplied as fixed blocks. Candidate intervals are ranked by physical span and SNP count, after which a non-overlapping set is retained. Tag SNPs are selected with a greedy LD-coverage algorithm. At each step, the variant covering the largest number of currently uncovered variants at or above a user-defined LD threshold is selected. Selection can be performed globally or independently within detected blocks. This provides a compact representative marker set for downstream assay design or summary analyses. The tag-SNP option is also related to LD-based selection of maximally informative markers (Carlson et al., 2004).

### 2.5 Derived analyses and subgroup comparisons

Additional functions identify LD neighbors of a focal variant, summarize LD decay in physical-distance bins, estimate haplotype frequencies across selected variants, calculate LD separately in arbitrary sample groups, and compute group-minus-reference LD matrices. A batch wrapper processes multiple genomic regions through the same one-call workflow. These analyses use the same linked ld_data and ld_result objects, allowing users to inspect intermediate objects, retain analysis history, and reuse results in custom R scripts.

### 2.6 Integrated regional visualization and export

The plot_ld function aligns one or two triangular LD heatmaps with optional GWAS association statistics, GFF3/GTF-derived gene models, allele-frequency tracks, block boundaries, tag SNPs, and user-highlighted variants. GWAS points can be colored according to r² with a lead variant, providing a local analogue of association-plus-LD regional plots. Gene tracks include transcript direction and available CDS/UTR structures. Output can be written as PDF, SVG, PNG, or TIFF. For ≤250 variants the default heatmap uses vector cells; above 250 variants the package automatically rasterizes only the heatmap layer while retaining full LD computation and tabular export. This strategy controls graphic-object complexity without sampling the statistical matrix. In addition to the R API, the package provides a one-call ldblockr workflow and a command-line interface. Common LDBlockShow-style flag aliases are accepted for users transitioning from an established command-line workflow. Exported tables include variant-level metadata, pairwise LD statistics, block definitions, tag SNPs, and LDBlockShow-style triangular compatibility files.

### 2.7 Validation design

Release validation was performed using the package’s bundled example data and official LDBlockShow version 1.41. The reference benchmark contains 60 diploid samples and 42 phased biallelic variants generated with three underlying LD segments, yielding 861 unique variant pairs. The same genotype content is distributed in phased VCF.GZ, HapMap, and numeric-matrix forms. Automated tests verify that VCF and HapMap readers recover identical genotype matrices and that the numeric-matrix and PLINK decoders produce the expected dimensions and genotype calls. Pairwise dosage r² is additionally checked against squared Pearson correlations computed directly in R. For external numerical cross-validation, all 861 pairs were analyzed with both LDblockR and LDBlockShow. Because LDBlockShow reports r² and D′ to three decimal places in its native output, agreement was assessed using a tolerance of 0.00051. The archived 2026-08–24 validation record reports a clean source installation and R CMD check --no-manual with zero errors and warnings on R 4.5.1 (R Core Team, 2025). A fresh build and check of the LDblockR 0.0.1 source archive supplied with this revision was run on Ubuntu 24.04 x86_64 with R 4.3.3; package installation, examples, and core tests passed with zero errors, but R CMD check reported one warning for duplicate Rd names/aliases. An empty pic/.keep placeholder that had produced an additional packaging note was removed from the supplied archive. The 2026-08–24 validation record predates this source snapshot.

### 2.8 Real crop-genomics case study: regional LD in the official maize TASSEL panel

The real-data example uses the supplied mdp_genotype.hmp.txt file, the HapMap-format genotype subset distributed in the official TASSEL tutorial data and bundled with rTASSEL (TASSEL Developers, 2026; rTASSEL data snapshot commit e8cbfcce47422319b5bfe8376cf5550ebd3b365e). The genotype file contains 281 taxa, 3,093 SNP markers, and AGPv1 coordinates. It represents a reduced marker subset of the historically important maize association-panel resource; the founding panel was assembled from diverse public-sector breeding germplasm, including temperate and tropical lines, sweetcorn, and popcorn (Flint-Garcia et al., 2005). The tutorial companion phenotype and population-structure files report 282 taxa, so they were not merged into this genotype-only LD case; no GWAS or phenotype association is claimed. This is a legacy, relatively sparse real-data workflow example; its marker coverage supports a workflow demonstration but does not provide dense local resolution. We decoded calls against the two declared marker alleles; 48 markers contained at least one non-missing call inconsistent with their declared allele pair and were excluded. All 3,045 remaining markers passed the missing-call fraction threshold of 0.20; filtering at MAF ≥ 0.05 retained 2,526 markers. Chromosome 1 contributed 428 retained SNPs and 91,378 unique dosage-r² pairs. Pairwise-complete sample sizes ranged from 201 to 281; median and mean r² were 0.00510 and 0.01277, and 42 pairs had r² ≥ 0.80. Median marker missingness on chromosome 1 was 2.49% (maximum 14.95%). For a locus-scale display, we selected the densest inclusive 1-Mb window (AGPv1 chromosome 1:267,615,478–268,615,477), which contains 16 SNPs and 120 pairs; this sparse interval is illustrative and is not used to infer a candidate gene or biological association. In this window, pairwise sample size was 226–277, median and mean r² were 0.0353 and 0.1054, and two pairs had r² ≥ 0.80. Pairwise dosage r² values independently calculated from the source HapMap calls matched the archived regional pair table to a maximum absolute difference of 5.01 × 10^−13^. The accompanying LDblockR R reproduction script was run successfully and generated the chromosome-1 and regional LD results, block/tag tables, PDF plots, and an RDS project object. The Python audit remains an independent numerical cross-check. Reproduction outputs and environment details are supplied with the benchmark materials.

### 2.9 Scalability benchmark design

Scalability was benchmarked with independent R processes using synthetic diploid dosage matrices. Marker-count tests held sample size at 281 and used 100, 250, 500, 1,000, 2,000, and 3,093 markers; sample-count tests held marker count at 500 and used 100, 281, 500, and 1,000 samples. Genotypes were simulated from marker allele frequencies drawn uniformly from 0.05–0.50, with 1% calls set missing. Each configuration was run three times. We recorded dosage-r² computation time, plotting time, output and R-object sizes, and process peak resident memory using GNU time. **Table 1** reports medians across replicates. This benchmark measures dosage-r² and regional heatmap rendering; it does not measure D′, block detection, GWAS, or compare against another package. The real maize workflow was also timed end-to-end as a separate data-specific demonstration.

**Table 1:** LDblockR dosage-r² benchmark across marker and sample dimensions.

Scaling series	Samples	Markers	LD computation (s)	Peak RSS (MiB)
Marker count	281	100	0.010	79.7
Marker count	281	250	0.052	141.4
Marker count	281	500	0.208	133.2
Marker count	281	1,000	0.871	147.7
Marker count	281	2,000	3.452	271.4
Marker count	281	3,093	8.138	479.9
Sample count	100	500	0.084	134.0
Sample count	281	500	0.199	133.2
Sample count	500	500	0.344	133.0
Sample count	1,000	500	0.696	132.3

Values are medians of three independent runs; peak RSS is for the complete R process.

## 3 Results

### 3.1 A unified R-native workflow and example use

The principal design outcome of LDblockR is the separation of genotype ingestion from downstream LD analysis. Once data are converted to ld_data, the same LD, block, plotting, and export functions can be applied regardless of whether the original data were VCF, HapMap, PLINK, or a matrix (**Figure 1**). This is particularly useful in crop-genomics projects in which historical HapMap datasets coexist with VCF- and PLINK-based workflows. The bundled test suite confirmed genotype equivalence between the VCF and HapMap encodings of the 60-sample, 42-variant benchmark. Native PLINK BED decoding was also verified with a small matrix containing homozygous, heterozygous, and missing genotypes. Unlike a plotting-only interface, the package retains intermediate analysis objects. Users can therefore obtain the genotype matrix, variant-level filtering statistics, pairwise LD matrices, block tables, tag SNPs, LD-decay summaries, subgroup results, and publication-ready graphics from the same scripted workflow. This object-based structure also enables downstream integration with user-defined R analyses without requiring intermediate text-file parsing. The release organization follows practical reproducible-computing and FAIR-data recommendations (Wilkinson et al., 2016; Wilson et al., 2017).

**Figure 1:**
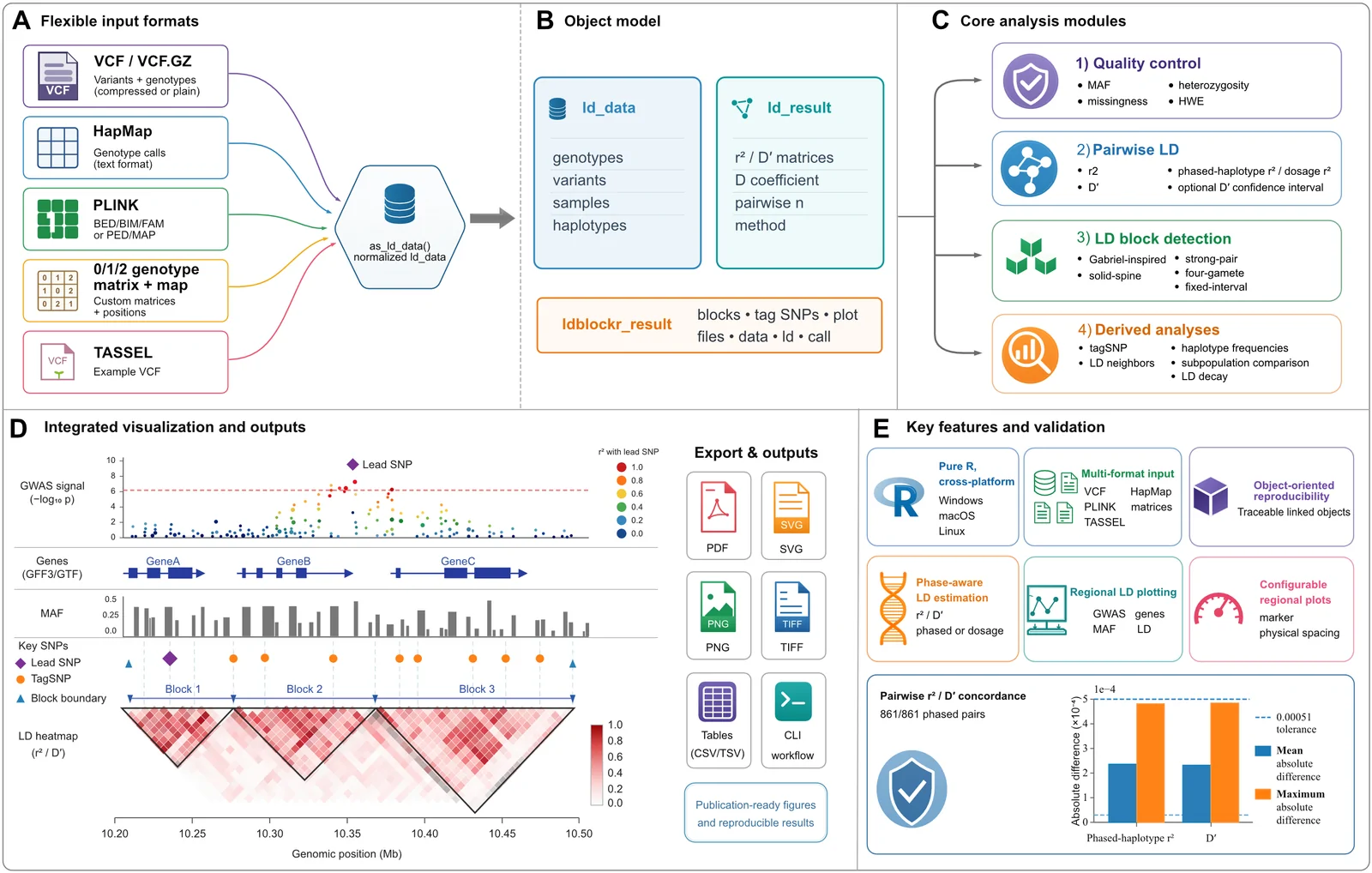
Overview of the LDblockR framework for reproducible linkage disequilibrium analysis and regional genomics visualization. **(A)** Supported input formats, including VCF/VCF.GZ, HapMap, PLINK, user-supplied 0/1/2 genotype matrices with marker maps, and the TASSEL example VCF. Input data are normalized into an ld_data object using as_ld_data(). **(B)** Object model of LDblockR. The ld_data object stores genotypes, variants, samples, and haplotypes; ld_result stores pairwise r²/D′ matrices, the D coefficient, pairwise sample size, and analysis methods; ldblockr_result organizes LD blocks, tag SNPs, plots, data, files, and analysis calls. **(C)** Core analysis modules, including quality control based on allele frequency, missingness, heterozygosity, and Hardy–Weinberg equilibrium; pairwise LD estimation using r² and D′ for phased-haplotype or dosage data; LD block detection using Gabriel-inspired, solid-spine, strong-pair, four-gamete, and fixed-interval strategies; and derived analyses including tag SNP selection, LD-neighbor analysis, haplotype-frequency analysis, subpopulation comparison, and LD decay. **(D)** Integrated regional visualization combining GWAS signals, gene annotations, allele frequency, key SNPs, LD block boundaries, and an LD heatmap, with outputs available in PDF, SVG, PNG, TIFF, table, and command-line workflow formats. **(E)** Key features and validation of LDblockR, including pure-R cross-platform implementation, multi-format input, traceable linked objects, phased- or dosage-based LD estimation, regional LD plotting, and configurable marker or physical-coordinate spacing. The lower panel shows numerical concordance of pairwise r² and D′ estimates, with 861/861 phased variant pairs within the 0.00051 tolerance threshold relative to LDBlockShow 1.41. In **(D)**, the displayed track is minor-allele frequency (MAF), as calculated and plotted by LDblockR; its range is 0–0.5.

Complete usage instructions, function references, built-in example-data descriptions, and manuscript-reproduction materials are available in the project repository at https://gitee.com/omixcore/LDblockR. The repository also provides the bundled examples and the scripts needed to reproduce the analyses reported in this article.

### 3.2 Numerical agreement with LDBlockShow

LDblockR reproduced the pairwise phased-haplotype r² and D′ values from LDBlockShow across all 861 unique SNP pairs. The maximum absolute difference was 4.993×1^−4^ for r² and 5.000×1^−4^ for D′, while the mean absolute differences were 2.349×1^−4^ and 2.372×1^−4^, respectively (**Table 2**). All 861 pairs for each statistic were within 0.00051 of the LDBlockShow output. Because the reference program reports these statistics to three decimal places in the compared output, these differences are consistent with output rounding and establish agreement at the reported precision.

**Table 2:** Numerical cross-validation of LDblockR against LDBlockShow 1.41.

Statistic	Pairs compared	Maximum absolute difference	Mean absolute difference	Pairs within 0.00051
Phased-haplotype r²	861	4.99 × 1^−4^	2.35 × 1^−4^	861 (100%)
D′	861	5.00 × 1^−4^	2.37 × 1^−4^	861 (100%)

### 3.3 Multiple block definitions and downstream regional analyses

The release tests exercised the Gabriel-inspired, solid-spine, threshold-based strong-pair, four-gamete, and fixed-interval block detection. These options allow the block definition to be matched to the analytical convention of a study rather than hard-coded to a single criterion. The same LD result can be reused to compare alternative block boundaries, which is important because block definitions are algorithm-dependent and should be reported explicitly in reproducible analyses. The test suite further verified greedy tag-SNP coverage, LD-neighbor extraction, haplotype-frequency normalization, distance-binned LD decay, and two-group LD comparison on the same benchmark object.

### 3.4 Integrated visualization and large-matrix rendering

LDblockR combines local association statistics and genomic annotation with the LD matrix in one figure. This supports a common post-GWAS task: moving from a lead association to the surrounding LD interval, inspecting candidate genes and correlated variants, and identifying block/tag structure without manually aligning independent graphics. The plotting system can display both r² and D′ panels, mark candidate SNPs, color association points by r² to the lead variant, show MAF, and draw gene models from GFF3/GTF. Large LD matrices create a separate visualization bottleneck even when the statistics can be computed. LDblockR therefore switches from vector cells to a rasterized triangular heatmap when the matrix contains more than 250 variants by default. An automated release test forced this path with 260 variants and confirmed successful raster output. Importantly, rasterization affects only the rendering layer; LD matrices and exported tables are not subsampled. Users can override the rendering mode and raster resolution.

These release tests also define the intended operating range: LDblockR is designed for candidate intervals containing tens to low thousands of variants, whereas whole-chromosome scans should be prefiltered with PLINK, BCFtools, or LDBlockShow before regional interpretation. This boundary reflects the approximately quadratic cost of pairwise D′ and block calculations, not subsampling of the exported statistical results.

### 3.5 Real maize case study: regional linkage disequilibrium in a public association panel

The maize tutorial subset provides a real genotype-data example with observed missing calls and allele-metadata conflicts. Following QC, the executed LDblockR workflow retained 2,526 markers from 281 taxa; the chromosome-1 analysis included 428 SNPs and 91,378 pairwise-complete dosage-r² estimates (n = 201–281 per pair). The median r² was 0.00510, with 42 pairs at or above 0.80, indicating a mostly weak but heterogeneous pattern among retained markers in this panel. The densest 1-Mb display window contains only 16 SNPs and 120 pairs; it is a visualization example rather than a biologically resolved candidate interval (**Supplementary Figure S1**). Independent Python calculations reproduced the archived regional pair estimates to within 5.01 × 10^−13^ maximum absolute difference. The full R reproduction script completed successfully in 10.49 s wall time with peak RSS of 160.3 MiB in the benchmark environment. This single end-to-end timing includes input parsing/QC, chromosome-1 and regional analyses, output generation, and plotting; it is specific to the tutorial data and hardware.

### 3.6 Scalability and intended operating range

In the marker-scaling series (281 samples), median dosage-r² computation increased from 0.010 s at 100 markers to 8.138 s at 3,093 markers; median peak RSS ranged from 79.7 to 479.9 MiB. In the sample-scaling series (500 markers), median computation time increased from 0.084 s at 100 samples to 0.696 s at 1,000 samples, while median peak RSS was 132.3–134.0 MiB. Each value is the median of three independent processes; **Table 1** gives the full series. The benchmark ran on Ubuntu 24.04.3 in a container with an AMD EPYC 9V74 CPU allocation (9 vCPUs), an 8-GiB memory limit, R 4.3.3, and LDblockR 0.0.1. Runtime and memory are environment-dependent. These measurements support regional dosage-r² computation for several thousand markers under the tested conditions, but do not establish performance for D′/block algorithms, GWAS, other hardware, or contemporary high-density genome-wide scans. LDblockR remains intended for focused regional interpretation; whole-genome preprocessing should use optimized tools such as PLINK, BCFtools, or LDBlockShow.

### 3.7 Functional comparison with existing LD workflows

LDblockR was designed as a complementary regional-analysis layer rather than a replacement for established LD software. LDBlockShow emphasizes fast command-line LD visualization from VCF and supports association/annotation tracks and subgroup analysis; PLINK 2 is optimized for large-scale genotype processing and LD calculation; Haploview provides an interactive framework for LD, haplotype blocks, and tag-SNP analysis; and LDheatmap provides flexible LD visualization inside R (Barrett et al., 2005; Purcell et al., 2007; Shin et al., 2006; Dong et al., 2021). LDblockR occupies a different practical niche by combining heterogeneous genotype input, reusable R-native objects, multiple block strategies, derived regional analyses, and integrated association/gene visualization in one scriptable workflow. **Table 3** therefore compares the packages by intended role rather than implying that established statistical procedures implemented in LDblockR are themselves novel.

**Table 3:** Practical positioning of LDblockR relative to established LD-analysis workflows.

Tool	Primary interface/common input	LD and block functions	Regional integration	Practical niche
LDblockR 0.0.1	R API + one-call workflow + CLI; VCF/VCF.GZ, HapMap, PLINK, 0/1/2 matrix	r², D′; five block strategies; tag SNPs; LD decay/neighbors; subgroup LD	GWAS + gene models + AF + highlighted variants + aligned LD heatmap	Reproducible candidate-region analysis across heterogeneous plant-genomics inputs
LDBlockShow	Compiled command line; primarily VCF	r²/D′ visualization and LD blocks; subgroup analysis	GWAS/statistics + GFF annotation + LD heatmap	Fast regional visualization and prefiltered large-data workflows
PLINK 2	High-performance command line; PLINK/VCF and related formats	Genome-scale pairwise LD, pruning/clumping and genotype processing	Limited publication-style gene/GWAS/LD integrated regional plotting	Genome-wide QC, filtering, association support, and scalable LD preprocessing
Haploview	Java GUI/command line; pedigree/HapMap-style data	r², D′, Gabriel-type blocks, haplotypes, tag SNPs	Interactive LD/block visualization; limited integrated gene/GWAS tracks	Interactive haplotype-block exploration and tag-SNP analysis
LDheatmap	R package; genotype/LD matrices	LD heatmap visualization; relies on external analyses for broader workflow	R-native LD heatmap with optional map/gene context; not a full post-GWAS workflow	Flexible R visualization when LD statistics and annotations are prepared separately

## 4 Discussion

LDblockR addresses a practical gap between high-performance command-line LD programs and the R environment in which many association-genetics analyses and figures are finalized. Its main contribution is workflow integration, interoperability, and reproducible regional visualization rather than a new statistical estimator. Several genotype representations are normalized once, after which established LD estimation, block calling, tag-SNP selection, subgroup comparison, LD decay, haplotype summaries, regional annotation, and plotting can be performed with a consistent object model. The numerical comparison with LDBlockShow provides an external check on the core pairwise statistics: all 861 phased benchmark pairs agreed at the three-decimal precision of the reference output. The attached maize HapMap subset adds real crop genotype data to the manuscript and exposes practical issues such as missing calls and allele metadata that may need QC before downstream analysis.

The biological context sets important limits on interpretation. The 3,093-marker HapMap file is a historical tutorial subset of a diverse public maize association resource, not a dense contemporary genome-wide panel. Its 281 taxa represent a structured breeding collection, so the observed r² distribution is specific to this panel and the retained markers; it should not be generalized as a species-wide LD estimate. The 1-Mb zoom contains only 16 SNPs, and no phenotype, association signal, candidate-gene annotation, or population-specific causal inference was analyzed here. Accordingly, the case is presented as a real-data genotype-QC and regional-LD workflow example, not a biological discovery; local interpretation is constrained by the marker coverage in this tutorial subset. The LDblockR R reproduction script was executed successfully to generate the case-study outputs, and the independent Python audit provides a separate numerical cross-check.

LDblockR intentionally prioritizes transparency and regional usability over maximum whole-genome speed. The r² matrix is efficiently derived from matrix correlations, but D′ estimation, D′ interval summaries, and several block algorithms require pairwise iteration and scale approximately quadratically with the number of variants. Our measured benchmark covers dosage-r² calculation and plotting only; D′, block detection, GWAS, and alternative tools were not benchmarked, so no speed advantage over optimized software is claimed. In the tested container, dosage-r² computation completed in a median of 8.138 s for 281 samples and 3,093 synthetic markers, with median process peak RSS of 479.9 MiB; a 281-sample/3,093-marker real-data workflow that computes chromosome-1 and selected regional LD completed end-to-end in 10.49 s with 160.3 MiB peak RSS. These machine-specific results do not imply whole-genome capacity or high-density locus resolution. For very large data sets, PLINK, BCFtools, or LDBlockShow should be used for genome-scale filtering or first-pass computation, followed by LDblockR for focused regional interpretation. Current limitations include restriction of the VCF reader to biallelic SNPs by default, lack of direct BGEN/PGEN support, no use of genotype likelihoods or dosage probabilities, and no parallel/C++ backend for pairwise D′ estimation. The PLINK BED reader currently requires SNP-major layout. Future development will focus on optional compiled/parallel backends, support for PGEN/BGEN and probabilistic dosages, additional block definitions, and richer interoperability with plant genomic annotation resources. Overall, LDblockR provides a reproducible, cross-platform R framework for regional linkage disequilibrium analysis that connects heterogeneous genotype inputs with phase-aware LD statistics, multiple haplotype-block definitions, tag-SNP and decay analyses, subgroup comparisons, genomic annotations, and publication-quality graphics.

## Data Availability

Publicly available datasets were analyzed in this study. This data can be found here: LDblockR source repository (version 0.0.1), including synthetic benchmark and TASSEL example files: https://gitee.com/omixcore/LDblockR.

## Supplementary material

Supplementary Figure S1 Real-data LD analysis using the official maize TASSEL association-panel tutorial data. **(A)** Lower-triangular heatmap of pairwise-complete dosage r^2^ for 428 post-QC SNPs on AGPv1 chromosome 1 across 281 inbred lines; 91,378 unique pairs were analyzed, with pairwise sample sizes of 201–281. The blue outline marks the densest 1-Mb window. **(B)** Zoom of that interval, containing 16 SNPs and 120 pairs (pairwise sample size 226–277). Before analysis, 48 of 3,093 markers with calls conflicting with their declared alleles were excluded; MAF ≥ 0.05 retained 2,526 markers genome-wide. Independent calculations from the source HapMap calls matched the archived 1-Mb pair table (maximum absolute difference 5.01 × 10^-13^).
